# The Use of Cone Beam CT(CBCT) in Differentiation of True from Mimicking Eagle’s Syndrome

**DOI:** 10.3390/ijerph17165654

**Published:** 2020-08-05

**Authors:** Miloš Tijanić, Nikola Burić, Kristina Burić

**Affiliations:** 1 Department of Oral and Maxillofacial Surgery, School of Medicine and Stomatology, University of Niš, Dr Zoran Đindjić Blvd. 52, 18000 Niš, Serbia; tijanicm@yahoo.com; 2 Intern, School of Stomatology, University of Niš, Dr Zoran Đindjić Blvd. 52, 18000 Niš, Serbia; kristinaburic@yahoo.com

**Keywords:** Eagle’s syndrome, panoramic radiography, CBCT acquisition, stylohyoid complex, collar-like

## Abstract

Differentiation of true from mimicking Eagle’s syndrome based on conventional radiography is difficult; however, cone beam computed tomography (CBCT) images can contribute to proper diagnosis of mimicking Eagle’s syndrome. The aim was to study radiological images of a 37-year old female patient (patient #1), with chronic cervicofacial pain who underwent radiological diagnosis with a conventional panoramic machine; another 75-year old male patient (patient #2), with chronic oropharyngeal pain, underwent a radiological diagnosis with the CBCT machine, with a field of a view of 16 × 12 cm. Exposure factors were 120 kVp, 7 mA, with a 20 s exposure time of acquisition. The results show a panoramic image (patient #1) with a pathologically elongated styloid process 46 mm of length, which was surgically removed, releasing the patient from further pain episodes. CBCT acquisition (patient #2) showed an impacted left maxillary canine in the edentulous maxilla and a peculiar elongation of both stylohyoid complexes as impressive, “collar-like”, bilateral, elongated, multiple segmented, calcified stylohyoid complexes, without pressure on the vital neurovascular neck structures, mimicking true Eagle’s syndrome. The impacted maxillary canine was surgically extracted with a subsequent resolution of pain episodes and the cessation of neurological complaints. The conclusions suggest that the use of CBCT images can contribute to differentiating mimicking from true Eagle’s syndrome, which has been rarely reported in the literature.

## 1. Introduction

Today, three dimensional (3D) cone beam computed tomography (CBCT), as a specialist medical tool, has become an important and inevitable source of 3D volumetric data in orofacial clinical practice, even though the initial purpose of CBCT was to perform angiography, mammography, and serve as guidance in radiotherapy [1]. CBCT can also serve to detect hidden or unclear anatomy, as well as occult maladies, thus reducing omissions in the early diagnosis of a relevant disease [2].

The first description of the pathologically elongated temporal styloid process was made by American ENT specialist, Dr. Watt Weems Eagle, in 1937 [3,4,5]. Eagle’s syndrome (styloid syndrome or styloid–carotid artery syndrome) is a rare condition where an elongated temporal styloid process (apex) is in proximity to the important neuro-vascular anatomical structures of the neck, causing pharyngodynia, and chronic head and neck pain (cervico-facial pain) [6]. The average normal length of the styloid process is 20–30 mm [7], projected to the lesser horns of the hyoid bone. The apex of the styloid process is interposed between the medially located internal carotid arteries and the laterally positioned external carotid arteries, and is in close proximity to the facial, accessory and vagus nerves, the internal jugular vein (medially located), and the glossopharyngeal nerve (under the styloid process), accounting for the glossopharyngeal neurological symptoms often observed in Eagle’s syndrome [7]. The base of the styloid process (tympanohyal), the styloid process itself (stylohyal), the stylohyoid/stylomandibular ligaments (ceratohyal) and lesser horn of hyoid bone (hypohyal) form the stylohyoid complex (SHC) [8,9], which is affected, and mineralized (ossified) in Eagle’s syndrome [3,4,5,7]. According to Fromer [10], embryological data has shown that the hyoid bone and styloid process, with its attached ligaments, originate from the first and second branchial cord, in addition to the cartilage structure, i.e., the so-called Reichert’s cartilage [11]. The stylohyoid complex can solidify during the course of life and be radiologically seen as an elongation, which is a more frequent variant, or as a complete ossified structure, which is a very rare variant; these abnormalities can cause pharyngodynia, which could be considered as pathognomonic for Eagle’s syndrome [12].

However, it is still clinically important to differentiate the true symptomatic Eagle’s syndrome and the asymptomatic elongated and calcified stylohyoid complex. Even though there is a plethora of reports of Eagle’s syndrome in the medical literature [3,4,5,8,10,11], only a few clinical cases of the “collar-like” symptom-free bilaterally elongated and calcified stylohyoid complex have been published [12].

It should be noted that the two-dimensional orthopantomographic technique has numerous shortcomings for radiographic analysis of anatomical structures and assessment of available bone tissue for various orofacial surgeries, primarily due to radiological enlargement of the anterior region of the mandible, possible distortions of the image, false image impression of enlarged anatomical structures, and overlapping of the close bony structure in the proximity of the styloid process [9]; all these shortcomings can lead to extremely erroneous radiological assessments of the true dimensions of the anatomical pathological structures of the oropharyngeal skeleton [13]. A radiological machine for computerized imaging of the jaws, which uses digital volumetric tomography (3D) with conical beam radiation (cone beam CT-CBCT), has shown superiority in detecting specific changes in the orofacial region, primarily due to low radiation dose, its exceptional precision and practicality, and the ability to make real-time radiological cross-sections of tissues in the axial, coronary, and sagittal planes, as well as panoramic cross-sections, cross-sections, oblique cross-sections, and temporomandibular joint imaging [14].

A dilemma in clinical practice is as follows: is it possible to use CBCT acquisition as radiological tool to help clinicians to differentiate a mimicking (false) from true, very rare oropharyngeal skeletal pathologies, i.e., a pathologically elongated temporal styloid process (Eagle’s syndrome).

Consequently, the aim of this paper is to present and describe the use of panoramic and CBCT images in differentiation of the true from the very rare, asymptomatic, “mimicking” Eagle’s syndrome patients.

## 2. Materials and Methods

This case study has been approved by the ETHICAL BOARD of the Clinic of Stomatology, Nis, Serbia, by its decision # 146/2-6. All procedures performed involving human participants were in accordance with the ethical standards of the institutional and/or national research committee, following the rules of the Declaration of Helsinki of 1975 (https://www.wma.net/what-we-do/medical-ethics/declaration-of-helsinki/), revised in 2013. We obtained patient’s consents at the time of admission at our department.

A 37-year-old woman (patient #1), was referred to our institution because of pain in the throat and the right side of the neck and face. Her pain episodes started four years ago, prior to planned mandibular teeth decay and root canal re-treatment of a tooth in the mandible and maxilla. She was constantly experiencing severe cervico-facial pain on the right side of the head, with the pain attacks lasting approximately five-ten minutes, and with a pain frequency of three-five times per day. The irradiation of pain was from the external auditory canal to the right side of the throat, with pain episodes during swallowing and moving tongue during speech. The patient had no past medical history significant for systemic or local disease, with the laboratory values of blood and urine within normal range.

**Figure 1:**
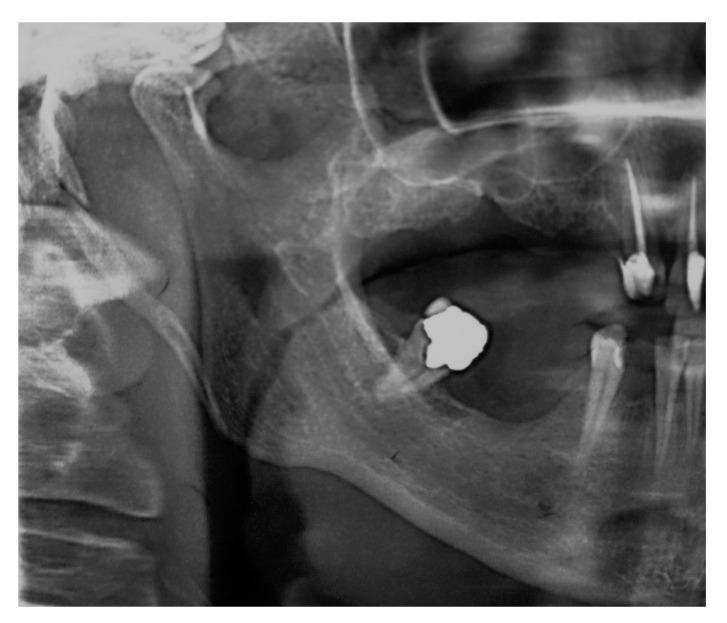
Cropped panoramic image showing pathologically elongated styloid process ending at the inner side of angle of mandible. The length of styloid process is 46 mm (conventional manual measurement with straight ruler).

The patient’s orofacial region was thoroughly examined with no clinical findings indicating the cause of her pain episodes. However, oropharyngeal examination showed severe tenderness and pain on palpation in the right tonsillar region, with motion pain occurrence during head horizontal rotation. Panoramic X-ray acquisition showed pathological elongation of the right styloid process, like a “thorn” with a length of 49 mm by manual measurement with a ruler (Figure 1).

A 72-year-old man (patient #2), was referred to our institution for the assessment of “inexplicable pain” in the left oropharyngeal region, with mild dysphagia and vague throat discomfort of a few minutes’ duration, first pain episodes having started two-three years before. The patient’s medical history was contributory for coronary heart disease and his laboratory data for calcium, vitamin D, and phosphorus metabolism were within normal range. Intraorally, avoiding the gag reflex, physical examination revealed that there was a palpable “asymptomatic foreign body” mass in the bilateral posterior alveololingual grooves. During the assessment of the patient’s edentulous left maxilla, intraoral clinical evidence of a possible unerupted maxillary canine emerged, barely showing the edge of the cusp of the maxillary canine. Panoramic X-ray acquisition was initially performed. However, in order to differentiate true from mimicking Eagles’ syndrome, additional CBCT scans were obtained using the 3DCT^®^ (from manufacturer MEDICA Systems and Products, Nis, Serbia), previously used successfully in the imaging of rare pathologies. The CBCT machine, 3DCT^®^, consists of an amorphous-silicon flat-panel detector, X-ray tube, and a source collimator. CBCT scans were performed in the patient’s sitting-up position, and with straps over the forehead in order to prevent movement of the head. During the gantry rotation of 360°, for the purpose of data acquisition, the mandible rested in a prefabricated chin cup. The technical parameters were standardized as follows: used field of view–16 (w) × 13 (h) cm, exposure time of 20 s, X-ray generator of 120 kVp with 7 mA, and a radiation dose of 37 micro-sievert (µSv). The obtained images were reconstructed by using the previously described algorithm and technical data which represents a combination of the high spatial frequency reconstruction algorithm and the acquired image data, consisting of a projection image matrix of 1560 × 1900 (pixels), with 14 bits that delivered 16,384 shades of gray for a better image contrast, by using an amorphous silicon digital X-ray imager (receptor), manufactured for the high-speed dental cone beam CT (PaxScan 2520D/CL; Varian Medical Systems, Inc., Salt Lake City, UT, USA); other technical parameters consisted of a volume matrix of 800 × 800 × 650 (voxels), volume size of 18 × 18 × 13 cm (i.e., 3615.840 cm^3^), and 0.3 × 0.3 × 0.3 mm (i.e., voxel size of 0.027 mm^3^). The image slices were reconstructed in the resolution mode, with the isotropic cubic voxel size of up to 300 µm (0.3 mm).

## 3. Results

**Figure 2:**
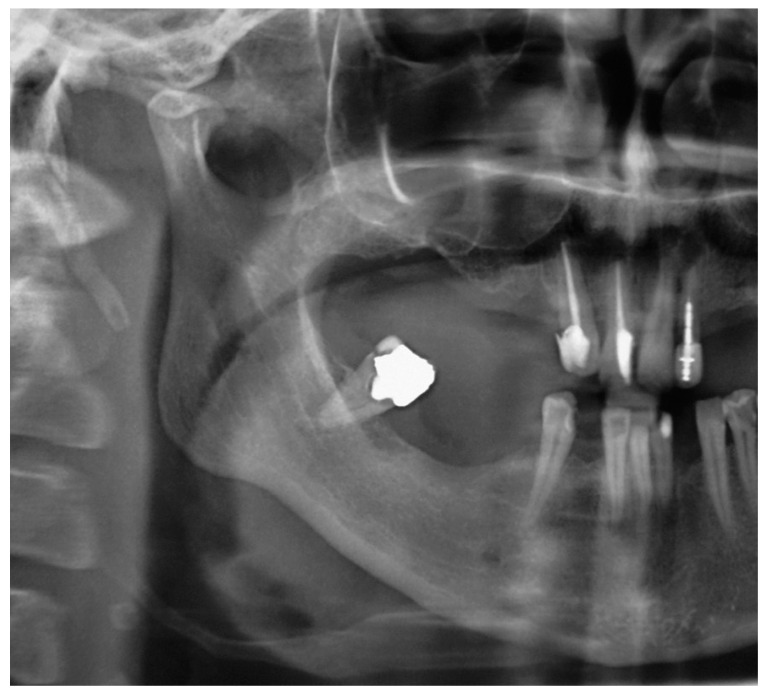
Cropped panoramic image showing resected styloid process.

Patient #1 is categorized in the group confirming diagnosis of true Eagle’s syndrome and scheduled for surgery. After preparation for general anesthesia, surgery was performed by the extraoral trans cervical per via approach into the parapharyngeal space, for identification of styloid process, which was subsequently surgically removed for half of its length (~25 mm), seen in repeated panoramic X-ray acquisition (Figure 2).

The postoperative course was uneventful and the patient was discharged from our institution on the seventh postoperative day. Over the next few weeks the patient stopped experiencing any pain in the right cervico-facial region with cessation of pain episodes.

**Figure 3:**
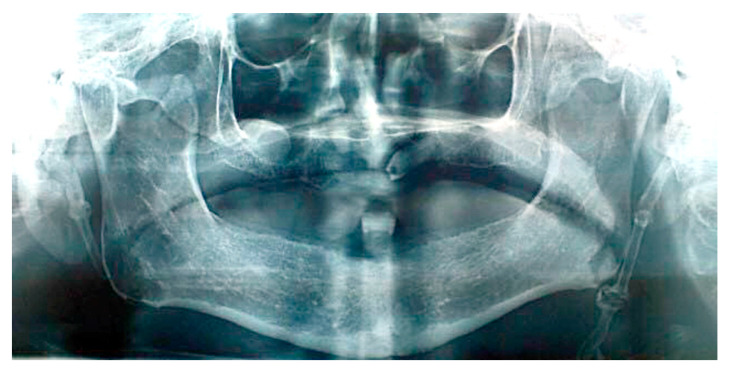
Panoramic X-ray acquisition showing bilaterally pathological nodular elongation of the left and right styloid processes extending beyond the lower border of the mandible; an unerupted impacted maxillary left canine and, on the right side, the sinus mucocele can also be seen.

Patient #2′s panoramic X-ray acquisition showed bilaterally pathological nodular elongation of the left and right styloid processes extending beyond the lower border of the mandible (Figure 3); an unerupted impacted maxillary left canine and, on the right side, the sinus mucocele was also observed. A tentative diagnosis was made that the patient had true Eagle’s syndrome due to the unusual panoramic image and pain experiences.

**Figure 4:**
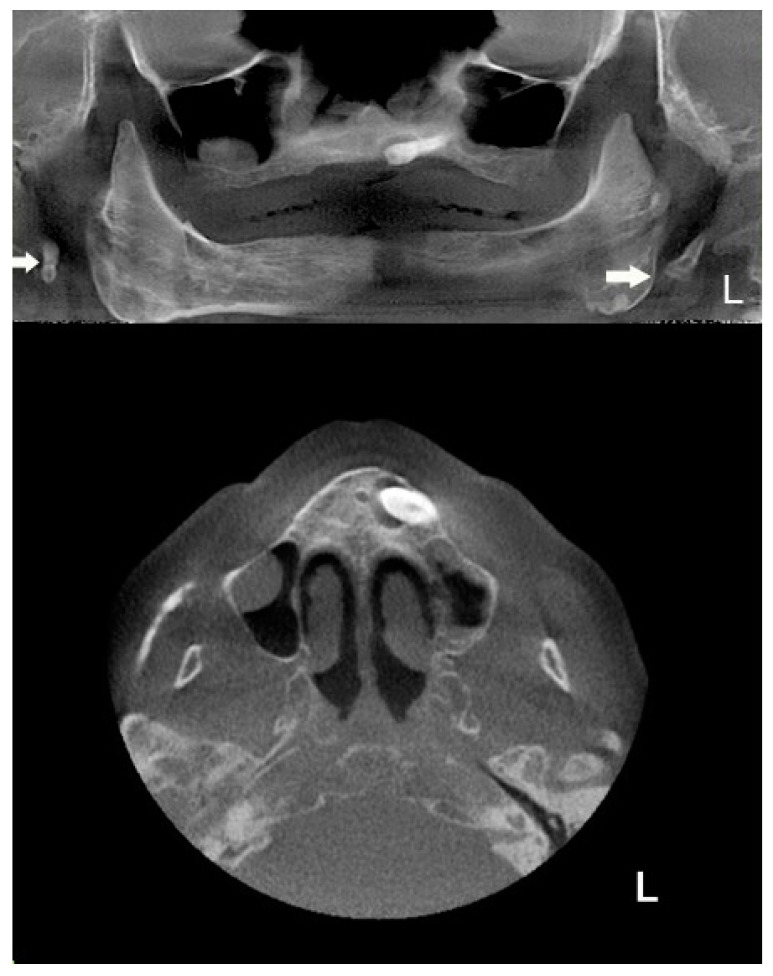
The axial cone beam computed tomography (CBCT) slice shows (**upper image**): panoramic radiography showing an unerupted maxillary left canine with a cyst-like formation around the canine crown and sinus pathology in the right maxillary sinuses. Elongated bilateral stylohyoid complex (SHC) processes are present and attain over one third of the mandibular ramus length (white arrows); (**lower image**): CBCT reconstruction images showing chronic inflammatory hyperplastic mucosa in the left maxillary sinus, impacted canine, while the right maxillary sinus exhibits a mucocele affecting the latero-basal portion of the sinus.

The extracted CBCT’s panoramic view shows an atypical bilateral elongation of the styloid process, with the unerupted impacted maxillary left canine and, on the right side, the sinus mucocele; the CBCT’s extracted panoramic and axial views of the maxilla shows a more detailed unerupted impacted maxillary left canine and two additional sinus pathologies in the maxillary sinuses; in the left maxillary sinus, there was chronic inflammatory hyperplastic mucosa, while the right maxillary sinus exhibited a sinus mucocele (Figure 4).

**Figure 5:**
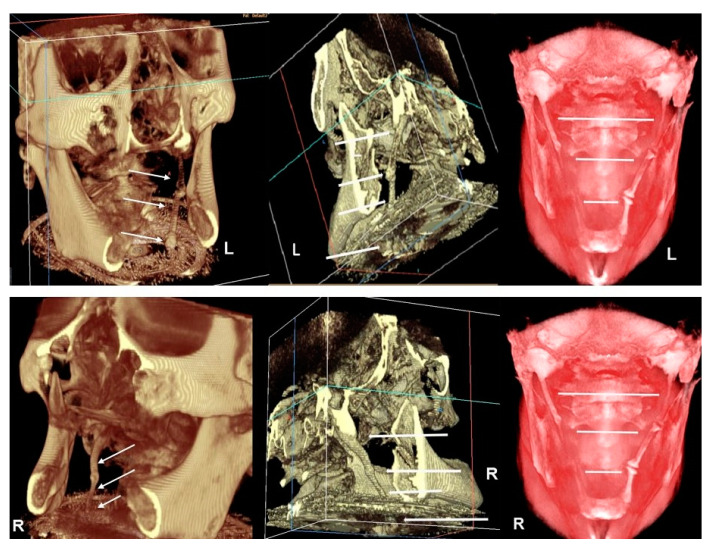
**Top image**–CBCT three-dimensional reconstruction shows an impressive calcification of the SHC in different image modes (left–default 2 mode, center–default mode, right-skin/red mode throughout antero-posterior slice sectioning). On the left side, the CBCT radiographic appearance of a “collar-like” and elongated SHC shows a multi-interrupted and segmented calcified SHC, fully stretched to the lesser cornu of the hyoid bone (see arrows). **Bottom image**–in a different image mode, on the right side, CBCT three-dimensional reconstruction shows the segmented calcified SHC which barely reaches the lesser cornu of the hyoid bone (left–default 2 mode, center–default mode, right–skin/red mode throughout antero-posterior slice sectioning) (see arrows).

Apart from these findings, there were multiple astonishing CBCT images of the different planes, which show an impressive bilateral mineralization of the styloid complex. All the taken CBCT radiographic images showed a “collar-like” multi-interrupted and segmented calcified stylohyoid complex (SHC). On the left side, the segmented calcified styloid ligaments fully stretched to the lesser cornu of the hyoid bone and on sagittal CBCT images, measuring 85.87 mm in length with an average width of 3.63 mm, while the right side barely reached the lesser horns of the hyoid bone, measuring 81.13 mm in length with an average width of 2.58 mm (Figure 5).

**Figure 6:**
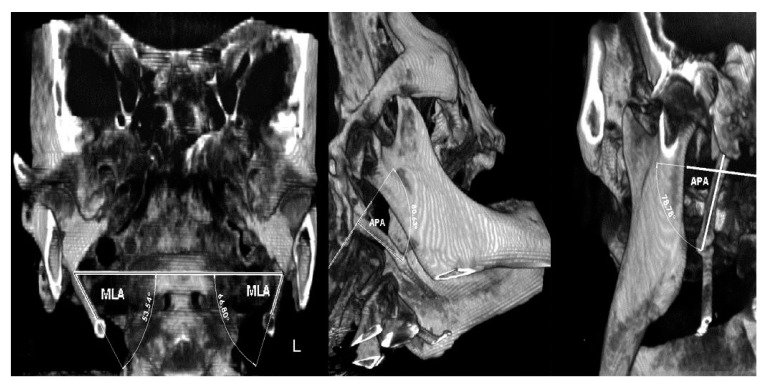
3D measurement of angles for analyzing a “normal-pathological” SHC: (**left**) measurements of mediolateral angling (MLA) of a calcified/ossified coronal 3D-CBCT image; (**middle**) measurements of right anteroposterior angling (APA) of a calcified/ossified SHC with the longitudinal axis of the SHC and Mc Rae’s line; (**right**) measurements of left anteroposterior angling (APA) of a calcified/ossified SHC with the longitudinal axis of the SHC and Mc Rae’s line.

Due to suspicion that this patient belongs to the Eagle’s syndrome group, additional angle measurements were performed. The measurements of important angles for 3D visualization of the SHC showed: right 53.54° (short) and left 66.8° (~normal) for the mediolateral angle (MLA), right 80.63° and left 78.78° (elongated) for the anteroposterior angle (APA), indicating wide both angles [9]. Measurements of maximum thickness with values of 4.56 mm and 3.6 mm (normal) for the right and left sides were seen, respectively. On both sides, the pattern of calcification/mineralization was almost identical, involving the tympanohyal, stylohyal, ceratohyal and hypohyal parts of the SHC (Figure 6).

In this clinical situation, we consider that pain of other origin, for example, dental pain from an impacted tooth, can contribute to this asymptomatic pain free patient with calcified styloid process, experiencing painful episodes, which can in fact be a “mimicking”, but not a symptomatic/painful true Eagle’s syndrome.

The patient was scheduled for the odontectomy of the impacted non-erupted left maxillary canine, which was surgically extracted. The patient experienced the complete resolution of previous cervicofacial pain episodes one month after odontectomy, with a subsequent cessation of further visits to our institution.

## 4. Discussion

There are numerous previously diagnosed reports of Eagle’ s syndrome in the literature [3,4,5,8,10,11,15], but 3-D CBCT images as an aid in differentiation of the bilateral asymptomatic (non-syndromic) “collar-like” calcified stylohyoid complexes from true, symptomatic (syndromic), painful Eagle’s syndrome are uncommon [16].

Pathological 3-D angulation and pressure towards the vital neuro-vascular neck structure of the elongated and mineralized SHC process is the paramount anatomical cause for “classic” painful Eagle’s syndrome [6].

It should be stressed, that the elongated styloid process that is mineralized is not pathognomonic for Eagle’s syndrome, because many patients with a mineralized, and elongated styloid process are asymptomatic-pain free patients [17].

In the panoramic view, when the styloid process is elongated, it attains over one third of the mandibular ramus length [18], which is presented on the panoramic image in patient #1. Taking all the obtained clinical facts into account (clinical symptoms and panoramic image) in the described case, diagnosis with proper subsequent surgical treatment of true Eagle’s syndrome was made in patient #1.

It is necessary to understand better and to clarify the importance of the origin of pain, which is an inseparable part in defining true Eagle’s syndrome. We define that a person with “mimicking/imitating Eagle’s syndrome” is a patient with calcified stylohyoid complex who experiences a pain of other origin (non-Eagle’s pain), which is not due to mineralized stylohyoid complex (SHC) [8,9], as a cause of SHC’s pressure on the neck’s nerve complexes; especially unilateral pain, which may came from another origin, such as an impacted maxillary canine in our case, which was in fact a contributing factor for “mimicking/imitating true (symptomatic, painful) Eagle’s syndrome.”

The diagnosis of “mimicking Eagle’s” syndrome is made due to the results of the 3–D left mediolateral angle (MLA) measurement, which showed a normal range on the left side with the value of 66.80° (~normal), with wide anteroposterior angle (APA), so we concluded by using 3–D CBCT visualization and MLA and APA measuring, that this case is “mimicking” but not a true Eagle’s case. The unilateral pain then must have come from another origin, which was an impacted maxillary canine in our case. The term “mimicking” is a literary term, which describes, in this case, a mimicking of a true painful Eagle’s syndrome with the pain origin due to pressure of the mineralized stylohyoid complex (SHC) on the neck’s nerves. Contributing to our proper diagnosis of mimicking Eagle’s syndrome is a pain free clinical course after surgical extraction of the maxillary impacted canine; all patient’s pain complaints disappeared within a few postoperative weeks, which means that there was no pressure on the vital neurological and vascular anatomic neck elements and it is more likely that the pain episodes were provoked and caused by the impacted maxillary canine.

The CBCT acquisition performed on patient #2 showed remarkable images of a segmented and completely calcified bilateral SHC, proving the necessity for CBCT imaging as an essential tool in the diagnosis of the cause of pharyngodynia, suspecting Eagle’s syndrome [17]. According to Langlais et al. [16], the pathological mineralization of the styloid complex is radiographically classified into three types. Type I is the elongated uninterrupted styloid process (more than 30 mm); type II is the pseudo-articulated styloid complex with stylo-mandibular and stylohyoid ligaments joined to the styloid process; and type III is the segmented and mineralized styloid complex. This classification could lead us to a diagnosis of the left type II and right type III mineralized styloid ligaments, in this case [15].

However, even though these CBCT radiographic images are impressive and support Eagle’s syndrome [19], it is still a debatable issue whether this is true Eagle’s syndrome or “mimicking” Eagle’s syndrome, due to the resolution of pharyngodynia (cervico-facial pain) after the surgical extraction of the impacted canine in patient #2. It is important to emphasize that some literature reports consider true Eagle’s syndrome only when the facial or neck pain originates from a SHC that is mineralized [20]. A pathologically elongated and mineralized SHC should be bonded with a symptomatic, palpable and painful “foreign body” mass in the throat, with constant chronic head and neck pain [6], due to the chronic irritation and pressure of the elongated styloid process on the vital neuro-anatomic elements in the neck, as in patient #1; these clinical findings were not discovered in their full capacity in patient #2. It is a known fact that orofacial pain is a pain within the trigeminal sensory neurological system incorporating also, among the oral anatomical structure, the pharynx and infratemporal fossa. Occurrence of micro movement of the impacted maxillary canine can be provoked by wearing a full denture, and subsequent local pericoronitis and its inflammation, as a trigger, contribute to subsequent irradiation of the pain stimulus via trigeminal nerve branches; all these pathological events can contribute in the presented case with calcified styloid process and impacted maxillary canine, to patient’s experience of pain, which can in fact be a “mimicking/imitating Eagle’s syndrome, but not a true Eagle’s syndrome”. There is a distinct possibility that a clinician, when faced with the impressive 3D “collar” like images of elongated stylohyoid complex/stalk extension, like the one we described, could firstly reach the tentative diagnosis of true Eagle’s syndrome without elaborating the possibilities, of other causes of unilateral cervicofacial pain, as was our case. That is why, in this patient with an impacted canine, we primarily surgically extracted this impacted canine and the patient in fact had clinically experienced various pain symptoms, which could trigger pain episodes manifested as sensory disturbances in the nasopharynx, mimicking pain like that in true Eagle’s syndrome. It is obvious that for these suspicious and unclear clinical cases, there is need for “active” 3-D radiological assessment of SHC and its angles, in order to make an accurate diagnosis of pain origin. Lengele and Dhem [20], suggested that a long styloid process with a downward, ventral and medial direction may be responsible for the creation of pain in true Eagle’s syndrome. Ramadan et al. [9] proposed several new parameters for analyzing the “normal vs. pathological” stylohyoid chain related to the mediolateral angle (MLA), the anteroposterior angle (APA), the angle of the styloid process’s base and the tip of the projection of SHC at the skull’s base bending, and the maximum thickness and length. Although the MLA and APA corners should show that there is a possibility of SHC pressure on the vital neurovascular neck structures with a consequent cervicofacial pain, which would support true Eagle’s syndrome, this has not been proved with CBCT visualization and measurement in our patient #2.

## 5. Conclusions

The presented cases of true and “mimicking” Eagle’s syndrome are good examples how the creation of obscure and rare pathological entities could be misdiagnosed or incompletely diagnosed with conventional 2-D radiological apparatus. CBCT can contribute to correct diagnosis of mimicking Eagle’s syndrome and the origin of pain episodes i.e., impacted canine in this case, showing numerous impressive images of the “collar-like” non-syndromic bilateral calcified stylohyoid complex, which has not been described often in the literature.

## References

[1] Scarfe W.C., Farman A.G. (2008). What is cone-beam CT and how does it work?. Dent. Clin. N. Am..

[2] Nakata K., Naitoh M., Izumi M., Inamoto K., Ariji E., Nakamura H. (2006). Effectiveness of dental computed tomography in diagnostic imaging of periradicular lesion of each root of a multirooted tooth: A case report. J. Endod..

[3] Eagle W.W. (1937). Elongated styloid process. Report of two cases. Arch. Otolaryngol..

[4] Eagle W.W. (1948). Elongated styloid process. Further observations and a new syndrome. Arch. Otolaryngol..

[5] Eagle W.W. (1949). Symptomatic elongated styloid process: Report of two cases of styloid process-carotid artery syndrome with operation. Arch. Otolaryngol..

[6] Politi M., Toro C., Tenani G. (2009). A Rare Cause for Cervical Pain: Eagle’s Syndrome. Int. J. Dent..

[7] Eagle W.W. (1962). The symptoms, diagnosis and treatment of the elongated styloid process. Am. Surg..

[8] Kawai T., Shimozato K., Ochiai S. (1990). Elongated styloid process as a cause of difficult intubation. J. Oral Maxillofac. Surg..

[9] Ramadan S.U., Gokharman D., Tunçbilek I., Kacar M., Kooar P., Kosar U. (2007). Assessment of the stylohoid chain by 3D-CT. Surg. Radiol. Anat..

[10] Frommer J. (1974). Anatomic variations in the stylohyoide chain and their possible clinical significance. Oral Surg. Oral Med. Oral Pathol..

[11] Prabhu L.V., Kumar A., Nayak S.R., Pai M.M., Vadgaonkar R., Krishnamurthy A., Madhan Kumar S.J. (2007). An unusually lengthy styloid process. Singap. Med. J..

[12] Desai V.D., Maghu S., Sharma R., Koduri S. (2014). Unique asymptomatic long bilateral calcified styloid process: A case report. J. Indian Acad. Oral Med. Radiol..

[13] Yim J.H., Ryu D.M., Lee B.S., Kwon Y.D. (2011). Analysis of digitalized panorama and cone beam computed tomographic image distortion for the diagnosis of dental implant surgery. J. Craniofac. Surg..

[14] Kamburoğlu K. (2015). Use of dentomaxillofacial cone beam computed tomography in dentistry. World J. Radiol..

[15] Hoffmann E., Räder C., Fuhrmann H., Maurer P. (2013). Styloid-carotid artery syndrome treated surgically with Piezosurgery: A case report and literature review. J. Cranio Maxillofac. Surg..

[16] Langlais R.P., Miles D.A., Van Dis M.L. (1986). Elongated and mineralized styholyoid ligament complex: A proposed classification and report of a case of Eagle’s syndrome. Oral Surg. Oral Med. Oral Pathol..

[17] Murtagh C.R.D., Caracciolo J.T., Fernandez T.G. (2001). Findings associated with eagle syndrome. Am. J. Neuroradiol..

[18] Chaves H., Costa F., Cavalcante D., Ribeiro T., Gondim D. (2013). Asymptomatic bilateral elongated and mineralized stylohyoid complex. Report of one case. Rev. Med. Chile.

[19] Moon C.H., Lee B.S., Kwon Y.D., Choi B.J., Lee J.W., Lee H.W., Yun S.U., Ohe J.Y. (2014). Eagle’s syndrome: A case report. J. Korean Assoc. Oral Maxilofac. Surg..

[20] Lengele B.G., Dhem A.J. (1998). Length of the styloid process of the temporal bone. Arch. Otolaryngol. Head Neck Surg..

